# Evaluation of a prototype metal artifact reduction algorithm for cone beam CT in patients undergoing radioembolization

**DOI:** 10.1038/s41598-024-66978-y

**Published:** 2024-07-16

**Authors:** Elif Can, Georg Böning, Willie Magnus Lüdemann, Clarissa Hosse, Johannes Kolck, Sophia Paparoditis, Thao Nguyen, Sophie K. Piper, Dominik Geisel, Gero Wieners, Bernhard Gebauer, Aboelyazid Elkilany, Martin Jonczyk

**Affiliations:** 1 Department of Diagnostic and Interventional Radiology, Charité – Universitätsmedizin Berlin, Corporate Member of Freie Universität Berlin and Humboldt Universität Zu Berlin, Augustenburger Platz 1, 13353 Berlin, Germany; 2 Institute of Biometry and Clinical Epidemiology, Charité – Universitätsmedizin Berlin, Corporate Member of Freie Universität Berlin and Humboldt-Universität Zu Berlin, Charitéplatz 1, 10117 Berlin, Germany; 3 Berlin Institute of Health at Charité – Universitätsmedizin Berlin, Charitéplatz 1, 10117 Berlin, Germany; 4 Institute of Medical Informatics, Charité – Universitätsmedizin Berlin, Corporate Member of Freie Universität Berlin and Humboldt-Universität Zu Berlin, Charitéplatz 1, 10117 Berlin, Germany; 5 Department of Diagnostic and Interventional Radiology, Medical Center University of Freiburg, Faculty of Medicine, University of Freiburg, Hugstetter Str. 55, 79106 Freiburg, Germany

**Keywords:** Radioembolization, Embolization, Metal artifact reduction, Cone beam computed tomography, Medical research, Outcomes research

## Abstract

Metal artifacts notoriously pose significant challenge in computed tomography (CT), leading to inaccuracies in image formation and interpretation. Artifact reduction tools have been designed to improve cone beam computed tomography (CBCT) image quality by reducing artifacts caused by certain high-density materials. Metal artifact reduction (MAR) tools are specific algorithms that are applied during image reconstruction to minimize or eliminate artifacts degrading CBCT images. The purpose of the study is to evaluate the effect of a MAR algorithm on image quality in CBCT performed for evaluating patients before transarterial radioembolization (TARE). We retrospectively included 40 consecutive patients (aged 65 ± 13 years; 23 males) who underwent 45 CBCT examinations (Allura FD 20, XperCT Roll protocol, Philips Healthcare, Best, The Netherlands) in the setting of evaluation for TARE between January 2017 and December 2018. Artifacts caused by coils, catheters, and surgical clips were scored subjectively by four readers on a 5-point scale (1 = artifacts affecting diagnostic information to 5 = no artifacts) using a side-by-side display of uncorrected and MAR-corrected images. In addition, readers scored tumor visibility and vessel discrimination. MAR-corrected images were assigned higher scores, indicating better image quality. The differences between the measurements with and without MAR were most impressive for coils with a mean improvement of 1.6 points (95%CI [1.5 1.8]) on the 5-point likert scale, followed by catheters 1.4 points (95%CI [1.3 1.5]) and clips 0.7 points (95%CI [0.3 1.1]). Improvements for other artifact sources were consistent but relatively small (below 0.25 points on average). Interrater agreement was good to perfect (Kendall’s W coefficient = 0.68–0.95) and was higher for MAR-corrected images, indicating that MAR improves diagnostic accuracy. A metal artifact reduction algorithm can improve diagnostic and interventional accuracy of cone beam CT in patients undergoing radioembolization by reducing artifacts caused by diagnostic catheters and coils, lowering interference of metal artifacts with adjacent major structures, and improving tumor visibility.

## Introduction

State-of-the-art angiography units feature flat-panel (FP) detectors mounted on C-arms for rotational acquisition of projection data. Similar to multidetector computed tomography (MDCT), cone beam computed tomography (CBCT) provides a 3D dataset, which can be reformatted to generate multiplanar reconstructions in various planes, enhancing imaging versatility^1^. CBCT can supplement fluoroscopy and digital subtraction angiography (DSA) for more precisely planning and guiding interventional procedures as it facilitates navigation of catheters or probes to the target lesion. In addition, CBCT allows onsite assessment of treatment outcome and identification of complications^2^.

FP detectors provide higher spatial resolution but lower soft tissue contrast than MDCT. With its low radiation dose, high versatility, and combination of fluoroscopy and tomography, CBCT is an optimal tool for transarterial radioembolization (TARE)^3–7^. In TARE, CBCT facilitates selective embolization prior to administration of the radiotherapeutic agent (yttrium-90-loaded microspheres)^8^.

However, strong artifacts caused by catheters, coils, and contrast agents can severely impair image quality and compromise decision-making during TARE based on CBCT images^9^. Metal artifacts are generally caused by photon starvation, beam hardening, or excessive quantum noise^10^. These nonlinear effects are amplified when a filtered back projection reconstruction algorithm is used and lead to dark and bright streaks radiating from the artifact source (metallic coil or catheter)^10–12^. Such streaks significantly impair image quality or obscure structures close to the artifact source, such as small vessels or adjacent hepatic parenchyma and tumors. However, these structures are often part of the target volume, and hence attenuation changes need to be clearly visible in order to assess the effect of embolization.

Metal artifact reduction (MAR) techniques can be employed to reduce such artifacts and improve the diagnostic yield of images^13^. MAR is applied in a second step after initial reconstruction of the CBCT raw data and usually takes a few seconds, depending on computer capacity. The main benefit of highly accessible CBCT is that it yields roadmaps supposed to make procedures simpler and safer while at the same time reducing the radiation dose^14^. Tomographic images help in the preparation, guidance, and execution of both intra-arterial and percutaneous interventions as well as in assessing the outcome of embolization. Thus, CIRSE/SIR advisory panels already recommend CBCT guidance in their protocol guidelines for various interventional oncologic procedures, such as transarterial chemoembolization (TACE) and TARE of the liver^15^.

The purpose of this study was to retrospectively assess the effect of an onsite MAR algorithm in CBCT on qualitative image parameters in patients undergoing TARE of malignant hepatic lesions.

## Materials and methods

### Study population

In this retrospective study, 40 consecutive patients (aged 65 ± 13 years; 23 men, 17 women) with liver metastasis (n = 18) or primary liver tumors (n = 22) undergoing evaluation for TARE including CBCT were enrolled from January 2017 through December 2018. An interdisciplinary team of oncologists, surgeons, and radiologists decided about acceptance of patients with predominant liver cancer not amenable to resection, local ablation, or systemic treatment for TARE. Inclusion criteria were: sufficient liver function and reserve, no or minimal extrahepatic manifestation of the tumor, and life expectancy of at least 12 weeks after TARE^14–17^. The study was approved by the local ethics committee (EA/187/15). All patients gave written informed consent.

### Image acquisition

A total of 45 CBCT examinations of the liver were performed using the same CBCT system (Allura FD 20, XperCT ND Roll protocol, Philips Healthcare, Best, The Netherlands). CBCT was performed during intra-arterial contrast agent injection into the celiac trunk using a split bolus injection protocol^18^. A diluted solution of Imeron® 300 [Bracco Imaging Deutschland GmbH, Konstanz, Germany] was prepared by adding the same amount of saline. The Accutron HP-D-HT® [Medtron, Saarbrücken, Germany] contrast injector was used for contrast agent administration through a 2.5F Cook Medical Cantata (Bloomington, USA) microcatheter. The first injection of 8 ml (1.5 ml/s) was performed to achieve presaturation of tumor parenchyma. 20 s following contrast agent administration, a second injection of 23 ml (1.5 ml/s) was administered. CBCT acquisition began 28 s after the start of the first injection.

CBCT was acquired at a rate of 31 images/s for 10 s using a peak tube voltage of 120 kVp. The acquired 3D volumetric CBCT images had an isotropic resolution of 0.6 mm. For extrahepatic branch embolization, coils (HILAL and TORNADO; Cook Medical, Bloomington, USA) were used. Projection data acquired were transferred to the postprocessing workstation (Philips Healthcare, Best, The Netherlands) for MAR reconstruction.

### Postprocessing and metal artifact reduction algorithm

The projection data were reconstructed using a standardized field of view (FOV) of 250 × 194 mm and a matrix size of 384 × 384 × 296 pixels at a default medium voxel size and standard liver kernel (smooth) for optimal soft tissue resolution. An automated MAR algorithm^19^ was applied to the reconstructed data.

### Qualitative assessment of CBCT with and without MAR

**Figure 1:**
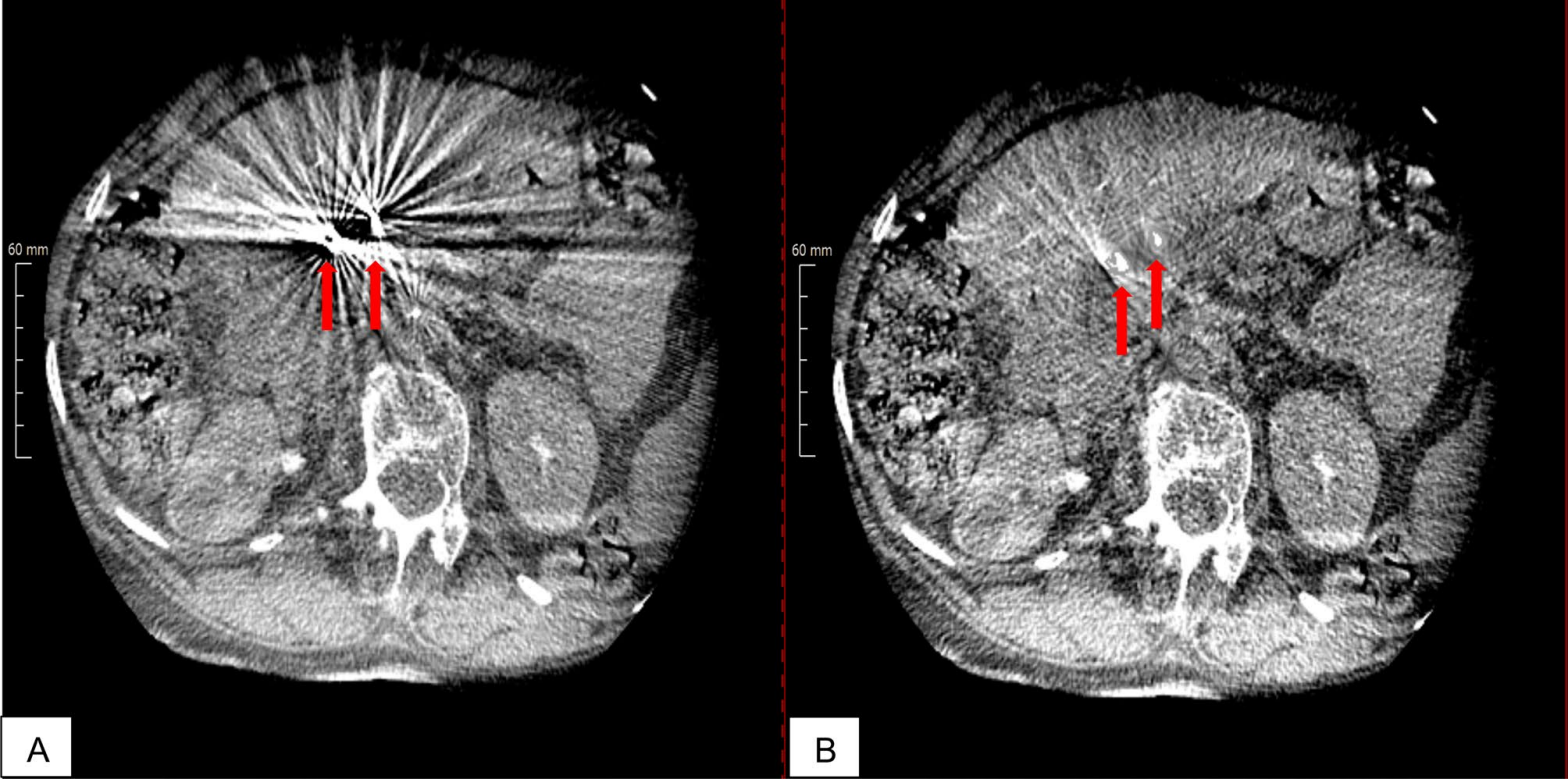
Illustration of the effect of MAR on CBCT images: shown is an image without MAR on the left (**A**) and an image with MAR on the right (**B**). The images were obtained in a patient with chronic hepatitis and hepatocellular carcinoma after coil embolization (13 × Boston Scientific and 4 × Cook, indicated by red arrows) in the gastroduodenal artery, left gastric artery, and accessory liver artery. Arrows indicate that there is marked artifact reduction after application of the MAR algorithm.

Qualitative evaluation of images with and without MAR was performed on regular clinical PACS workstations. Axial slices of the multiplanar reconstructions (MPRs) were presented in a side-by-side display of uncorrected and MAR-corrected images. An example of image presentation is shown in Fig. 1. Qualitative assessment was performed by four readers (interventional radiology fellows with 6 years (Rater 1), 3 years (Rater 2), and 1 year (Rater 3) of experience and 1 reader with no experience in interventional radiology (Rater 4). The readers scored artifacts caused by coils (most frequently in the gastroduodenal and right gastric artery), diagnostic catheters, surgical clips, or stents in the common hepatic duct (CHD) in uncorrected and MAR-corrected images using a five-point Likert scale (1 = artifacts affecting diagnostic information to 5 = no artifacts).

In addition, readers scored visualization of the renal collecting system with often persisting contrast agent (1 = unacceptable to 5 = excellent visualization), tumor visibility (1 = artifact mimicking a lesion to 5 = clearly depicted lesion), and vessel discrimination (1 = unacceptable to 5 = excellent vessel discrimination) in images without and with MAR. Finally, motion artifacts were scored using a 5-point scale (1 = unacceptable to 5 = excellent visualization).

### Statistical analysis

Statistical analysis was performed using SPSS software (IBM; New York, USA), DATAtab e.U. Graz, Austria (2023.) and R software. Sample size was calculated with a two-group t-test of equivalence in means using nQuery Advisor V. 7.0 at a significance level of α = 0.05 and a desired power of 0.8. Descriptive statistics are given as mean with 95% confidence interval (CI), median with limits of the interquartile range (IQR, 25th and 75th percentile), minimum and maximum as well as absolute and relative frequencies, depending on scale.

Improvement in Likert scores with MAR algorithm compared to without were calculated with 95% confidence intervals for each artifact source and each reader. Moreover, averages over all 4 raters were calculated with 95% confidence intervals.

Kendall’s W coefficient of concordance was determined for interrater variability. Agreement between 0.81 and 1.00 was rated as perfect, between 0.61 and 0.80 as good, between 0.41 and 0.60 as moderate, between 0.21 and 0.40 as fair, and less than 0.20 as poor^20,21^.

### Ethics approval and consent to participate

All procedures performed in studies involving human participants were in accordance with the ethical standards of the institutional and/or national research committee and with the 1964 Helsinki declaration and its later amendments or comparable ethical standards. This article does not contain any studies with animals performed by any of the authors. The study was approved by the local ethics committee (EA/187/15-Ethikkommission der Charité—Universitätsmedizin Berlin). Informed consent was obtained from all individual participants included in the study.

## Results

**Figure 2:**
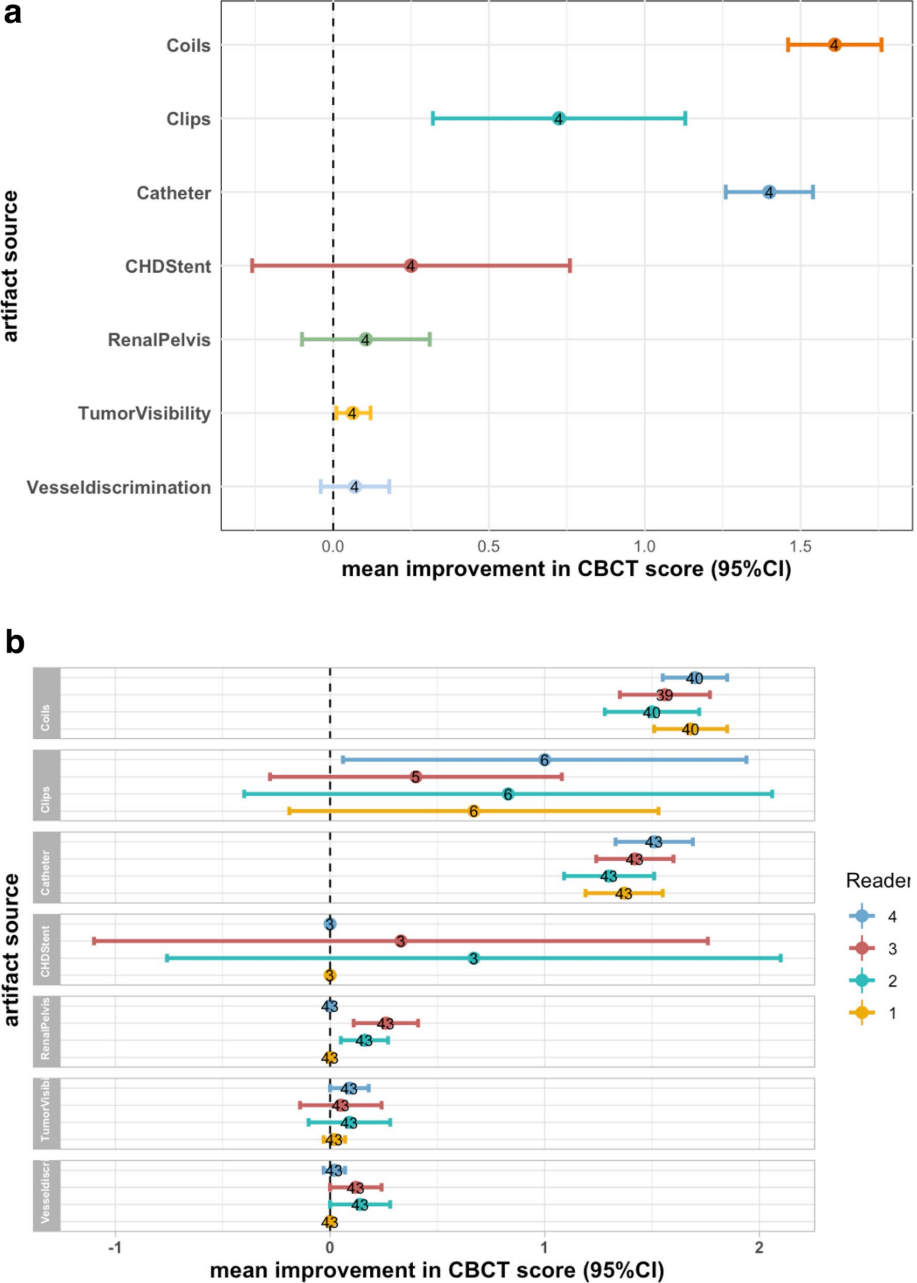
Forest plots of mean improvement in CBCT measurements with MAR compared to and without MAR for all 4 readers (**a**) and mean individual measurements for each reader (**b**).

Overall, MAR led to higher ratings, indicating better image quality. The differences between the measurements with and without MAR were most impressive for coils with a mean improvement of 1.6 points (95%CI [1.5–1.8]) on the 5-point likert scale, followed by catheters 1.4 points (95%CI [1.3–1.5]) and clips 0.7 points (95%CI [0.3–1.1]). Improvements for other artifact sources were consistent but relatively small (below 0,25 points on average) (Tables 1, 2, Fig. 2).

**Table 1:** Mean, median, 95% confidence intervals (CI), and IQR of the difference between CBCT with and without MAR for each artifact source and each reader. Results for motion as artifact source are not listed due to missing data for MAR-corrected images.

		Nr. of cases	Mean difference	95% CI of Mean		Median difference	25th Perc	75th Perc	Min Diff	Max. Diff
				Lower limit	Upper limit					
Coils	Reader 1	40	1.68	1.51	1.85	2.0	1.00	2.00	0	2
	Reader 2	40	1.50	1.28	1.72	2.0	1.00	2.00	0	2
	Reader 3	39	1.56	1.35	1.77	2.0	1.00	2.00	0	2
	Reader 4	40	1.70	1.55	1.85	2.0	1.00	2.00	1	2
Clips	Reader 1	6	0.67	− 0.19	1.53	0.5	0.00	1.00	0	2
	Reader 2	6	0.83	− 0.40	2.06	0.5	0.00	1.00	0	3
	Reader 3	5	0.40	− 0.28	1.08	0.0	0.00	1.00	0	1
	Reader 4	6	1.00	0.06	1.94	1.0	0.25	1.75	0	2
Catheters	Reader 1	43	1.37	1.19	1.55	1.0	1.00	2.00	0	2
	Reader 2	43	1.30	1.09	1.51	1.0	1.00	2.00	0	3
	Reader 3	43	1.42	1.24	1.60	1.0	1.00	2.00	0	2
	Reader 4	43	1.51	1.33	1.69	2.0	1.00	2.00	0	3
CHD stents	Reader 1	3	0.00	0.00	0.00	0.0	0.00	0.00	0	0
	Reader 2	3	0.67	− 0.76	2.10	1.0	0.50	1.00	0	1
	Reader 3	3	0.33	− 1.10	1.76	0.0	0.00	0.50	0	1
	Reader 4	3	0.00	0.00	0.00	0.0	0.00	0.00	0	0
Renal pelvis	Reader 1	43	0.00	0.00	0.00	0.0	0.00	0.00	0	0
	Reader 2	43	0.16	0.05	0.27	0.0	0.00	0.00	0	1
	Reader 3	43	0.26	0.11	0.41	0.0	0.00	0.00	0	2
	Reader 4	43	0.00	0.00	0.00	0.0	0.00	0.00	0	0
Tumor visibility	Reader 1	43	0.02	− 0.03	0.07	0.0	0.00	0.00	0	1
	Reader 2	43	0.09	− 0.10	0.28	0.0	0.00	0.00	− 2	2
	Reader 3	43	0.05	− 0.14	0.24	0.0	0.00	0.00	− 2	1
	Reader 4	43	0.09	0.00	0.18	0.0	0.00	0.00	0	1
Vessel discrimination	Reader 1	43	0.00	0.00	0.00	0.0	0.00	0.00	0	0
	Reader 2	43	0.14	0.00	0.28	0.0	0.00	0.00	− 1	2
	Reader 3	43	0.12	0.00	0.24	0.0	0.00	0.00	0	2
	Reader 4	43	0.02	− 0.03	0.07	0.0	0.00	0.00	0	1

**Table 2:** Average Mean Differences in CBCT Images with and without MAR, by Artifact Category, Including 95% Confidence Intervals (Across All Four Readers).

Method	Mean	95% CI	
		Lower	Upper
Coils	1.6100	1.46	1.76
Clips	0.7250	0.32	1.13
Catheter	1.4000	1.26	1.54
CHD stent	0.2500	− 0.26	0.76
Renal pelvis	0.1050	− 0.10	0.31
Tumor visibility	0.0625	0.01	0.12
Vessel discrimination	0.0700	− 0.04	0.18

However, the differences between the measurements with and without MAR regarding vessel discrimination and tumor Visibility were not significant, with a mean improvement of 0.07 points (95%CI [− 0.04–0.18]) and 0.06 points (95%CI [0.01–0.12]), respectively. This could be explained by the overall lower diagnostic imaging quality of CBCT (Tables 1, 2, Fig. 2).

### Inter-rater agreement

Apart from cases with CHD clips (in uncorrected images) and CHD stents (both MAR-corrected and uncorrected images), interrater agreement was good to perfect (Kendall’s W coefficient = 0.68–0.95) (Table 3). Use of the MAR technique led to higher Kendall’s W coefficients in most cases, indicating higher diagnostic accuracy. Specifically, differences in Kendall’s W coefficients were observed for clips (0.86 with MAR vs 0.18 without MAR), coils (0.80 with MAR vs 0.68 without MAR), and catheters, (0.86 with MAR vs 0.71 without MAR). Conversely, the differences in Kendall’s W coefficient for CHD stent were small and not significant (0.38 with MAR vs 0.19without MAR). For CHD-stents, the difference in Kendall’s W was of similar strength but agreement was generally low (0.38 with MAR vs 0.19 without MAR). Overall, the findings suggest that the use of the MAR technique can improve the overall diagnostic accuracy, especially for certain types of procedures.

**Table 3:** Kendall's W Coefficient of Concordance for Evaluating Interrater Variability. Concordance ratings are categorized as follows: 'Perfect' for values between 0.81 and 1.00, 'Good' for 0.61 to 0.80, 'Moderate' for 0.41 to 0.60, 'Fair' for 0.21 to 0.40, and 'Poor' for values below 0.20. Note: Data for motion artifact with MAR is not included due to its unavailability; only information for motion without MAR is provided.

Variables		W	95% CI	
			Lower	Upper
Coils	NoMAR	0.68	0.66	0.89
	MAR	0.80	0.80	0.94
Clips	NoMAR	0.18	0.10	0.87
	MAR	0.86	0.80	1.00
Catheter	NoMAR	0.71	0.71	1.00
	MAR	0.86	0.84	0.97
CHD stent	NoMAR	0.19	0.06	0.81
	MAR	0.38	0.25	0.81
Renal pelvis	NoMAR	0.89	0.89	0.96
	MAR	0.85	0.83	0.97
Tumor visibility	NoMAR	0.86	0.85	0.98
	MAR	0.96	0.95	0.98
Vessel discrimination	NoMAR	0.90	0.90	0.96
	MAR	0.95	0.94	1.00
Motion	NoMAR	0.84	0.84	0.98

## Discussion

Metal artifacts notoriously pose significant challenge in CT, leading to inaccuracies in image formation and interpretation^23^. Artifact reduction tools have been designed to improve CBCT image quality by reducing artifacts caused by certain high-density materials. Metal artifact reduction (MAR) tools are specific algorithms that are applied during image reconstruction to minimize or eliminate artifacts degrading CBCT images^24^.

The severity of raw data corruption depends on the volume and geometry of a metal object. For instance, one or more metallic embolization coils deposited in small vessels cause heavier corruption of raw projection data than a straight aligned catheter. In addition, metal constituents of coils, usually platinum with a high density and therefore high attenuation, may cause more severe artifacts than catheters. Factors contributing to metal artifacts include beam hardening, scatter, noise, photon starvation, and edge effects. These factors can corrupt the data and lead to inaccurate representations of tissues near the metal during reconstruction. As a result, analysis of tissue close to metal within the body might be unreliable or even impossible, depending on the amount and composition of the metal present^25^.

Cone beam CT is used in a wide range of clinical settings, including image-guided radiation therapy, image-guided interventions, and diagnostic applications requiring dedicated scanners such as breast, dental, and extremity imaging^26^.

Recent advances in MAR technology have been shown to contribute to the reduction of metal artifacts in conventional MDCT by using monoenergetic extrapolation from dual-energy or iterative reconstruction from single-energy datasets^27^. MAR algorithms in CBCT have already been investigated in the field of interventional neuroradiology and have been proven to increase visibility of hemorrhage, brain parenchyma, and vessels after stent or coil placement^9,28^. The segmentation algorithm automatically detects the metal trace in the raw data, which simplifies handling of the algorithm without any presets^29,30^.

In patients undergoing TARE, detection of aberrant vessels, residual vascularization of gastrointestinal organs, or extrahepatic reflux is necessary to prevent adverse effects such as duodenal or gastric ulcers. Therefore, high-resolution and accurate imaging of hepatic vasculature, including aberrant vessels, is essential. However, image quality of CBCT can be significantly compromised by metal-induced artifacts from intraprocedural catheters, previously placed coils for embolization of vessels, or high levels of intravascular contrast agent. An efficient and straightforward onsite implementation of this technique would greatly enhance the diagnostic and therapeutic value of CBCT.

The current retrospective study of a prototype metal artifact reduction (MAR) algorithm in patients who underwent CBCT for radioembolization showed that use of MAR significantly reduced coil- and catheter-induced artifacts and, to a lesser degree, also artifacts induced by other materials such as stents in the CHD.

The high interrater agreements we found in the present analysis—especially the higher agreement seen for MAR-corrected images—indicate that qualitative scores and image characteristics are reproducible. The MAR algorithm works for different kinds of artifact sources, in the present study we found the most pronounced effect for artifacts caused by angiographic catheters and metallic coils. Qualitative assessment showed significant improvement in terms of artifact reduction and visibility of blood vessels and liver parenchyma.

The current study has several limitations. The retrospective study design which might introduce inherent limitations, particularly related to patient selection bias and the subjectivity in image analysis. While our findings demonstrate the efficacy of the MAR algorithm in CBCT during TARE, our analysis was retrospective, focusing on qualitative parameters, while we did not prospectively investigate its influence on everyday clinical decisions.

Furthermore, the qualitative assessment of image quality by different readers is subjective and might introduce inter-reader variability, which we attempted to mitigate through interrater agreement analysis. In the present study, we reported a good to perfect interrater agreement, yet subjective scoring by readers still poses a possible risk of variability. To address this, future studies should incorporate quantitative metrics for image quality assessment. Quantitative measures can provide a more objective and reproducible evaluation of image quality improvements introduced by the MAR algorithm which might be specifically relevant to further validate the clinical impact of the MAR algorithm on decision-making as well as improving procedural accuracy, patient safety and patient outcomes.

In addition, the present study was conducted using a specific CBCT system and MAR algorithm, which may limit the generalizability of the findings. Different CBCT systems and MAR algorithms might yield varying results. Therefore, comparative prospective studies across multiple platforms are necessary to determine the efficacy of different MAR techniques universally. This would ensure that our findings are applicable to a wider range of clinical practices and imaging technologies.

Nevertheless, it is plausible to suggest that MAR correction contributes to clearer image interpretation, potentially saving time and minimizing radiation exposure as well as enhancing diagnostic confidence^29,30^.

Another limitation is the relatively small and heterogenous study population, comprising patients with various types of metallic coils and diagnostic catheters, each contributing differently to image artifacts, which may limit the generalizability of the findings. Further prospective studies with larger patient cohorts are necessary to validate these results and ensure they are applicable across diverse clinical settings in order to provide a more comprehensive understanding of MAR techniques and their diagnostic and therapeutic impact.

Additionally, while improved visualization of contrast agent in gastrointestinal organs due to MAR might prevent residual vascularization or extrahepatic reflux, this aspect was not addressed in the present study. Furthermore, all patients were examined using a standard CBCT protocol for abdominal interventions. Protocols with higher radiation doses might further diminish metal artifacts, though MAR techniques aim to reduce these artifacts without increasing radiation exposure.

## Conclusion

In conclusion, use of a metal artifact reduction (MAR) algorithm enhances diagnostic and interventional precision in cone beam CT when used in patients undergoing radioembolization. Such an algorithm effectively reduces artifacts caused by diagnostic catheters and coils, minimizes the interference of metal artifacts with adjacent critical structures, and improve tumor visibility.

## Data Availability

The datasets used and/or analyzed during the current study are available from the corresponding author on reasonable request.

## Abbreviations

CT: Computed tomography
CBCT: Cone beam computed tomography
MAR: Metal artifact reduction
TARE: Transarterial radioembolization
FP: Flat-panel
MDCT: Multidetector computed tomography
DSA: Digital subtraction angiography
TACE: Transarterial chemoembolization
